# The effectiveness of web-based and face-to-face continuing education methods on nurses' knowledge about AIDS: a comparative study

**DOI:** 10.1186/1472-6920-9-41

**Published:** 2009-07-10

**Authors:** Alireza Khatony, Nahid Dehghan Nayery, Fazlolaah Ahmadi, Hamid Haghani, Katri Vehvilainen-Julkunen

**Affiliations:** 1 PhD Department, School of Nursing & Midwifery, Tehran University of Medical Sciences, Tehran, Iran; 2 Postgraduate Department, School of Nursing & Midwifery, Tehran University of Medical Sciences, Tehran, Iran; 3 Nursing Department, Tarbiat Modares University, Tehran, Iran; 4 Statistics Department, School of Management and Medical Information & Health Sciences, Iran University of Medical Sciences, Tehran, Iran; 5 Nursing Department, University of Kuopio, Kuopio, Finland

## Abstract

**Background:**

Information about web-based education outcomes in comparison with a face-to-face format can help researchers and tutors prepare and deliver future web-based or face-to-face courses more efficiently. The aim of this study was to compare the effectiveness of web-based and face-to-face continuing education methods in improving nurses' knowledge about AIDS.

**Methods:**

A quasi-experimental method was used with a pre-test and post-test design. In this study 140 nurses with BSc degrees were chosen through a random sampling method and divided into a web-based and a face-to-face group by random allocation. For the former group the intervention consisted of a web-based course on AIDS; the latter received a 3-hour lecture course on the same subject. At the beginning and end of the course in both groups, the nurses' knowledge was measured by a questionnaire. Pre- and post-test scores were compared within and between the groups.

**Results:**

The results show that there was no significant difference between the groups in either the pre-test (t_(138) _= -1.7, *p *= 0.096) nor the post-test (t_(138) _= -1.4, *p *= 0.163) scores in the knowledge test. However, there was a significant difference in the pre-test and post-test scores within each group (web-based, *t*_(69) _= 26, *p *< .001; face-to-face, *t*_(69) _= 24.3, *p *< .001).

**Conclusion:**

The web-based method seems to be as effective as the face-to-face method in the continuing education of nurses. Therefore, the web-based method is recommended, as complementary to the face-to-face method, for designing and delivering some topics of continuing education programs for nurses.

## Background

Continuing education programmes have an important role in staff development [1,2]. In the nursing profession the most common aims of these programmes are to enable nurses to continue their professional growth, deliver safe and efficient care, appraise clinical practice in an innovative way and identify their own educational needs [2]. Research shows that continuing education leads to increased productivity, fewer occupational accidents, an improved organizational atmosphere and enhanced quality care [3]. Descriptive studies have also emphasized the importance of access to continuing education as an effective factor in reducing professional isolation, increasing the willingness to work in remote locations and improving staff performance in these areas [4].

Studies show that nurses strongly desire to participate in continuing education programs and the number of requests for participation is increasing [2,5]. Currently the most frequently used continuing education method in Iran is the face-to-face method. However, studies show that this method has several limitations; it does not recognize personal differences and learners' needs, and in some cases disregards problem-solving, creative thinking and other high-level cognitive skills[6]. On the other hand, Fordis (2005) criticized continuing education for being episodic and producing a "one-size-fits-all" format. He believed that improving the effectiveness of current continuing education delivering methods is crucial [7]. Many scholars have also emphasized the necessity of changing or complementing face-to-face methods for continuing education [8]. New advances in information technology provide improved opportunities for continuing education for nurses particularly via the web [6]. Teaching through the web can overcome some of the limitations of face-to-face teaching methods and provides easy and adaptable access to learning [9]. A web-based method can be used as an alternate mode of teaching and learning and as a substantial supplement to traditional teaching methods [6].

By using the web, different learning styles can be taken into consideration, distance learning becomes possible, more time is saved, and information and skills can be shared [6,7]. Studies of nurses' experiences of web-based learning have shown increased learner satisfaction, activeness in learning, ease of access, as well as a desire to use technology for learning [9-11].

Academic curriculum planners in Iran have paid attention to the issue of information technology and its application in different aspects of education in recent years. One example is the establishment of an electronic education center in Tehran University of Medical Sciences in 2002 to develop and evaluate electronic courses in order to improve education quality, promote the application of new educational technologies, offer tele-education courses and conduct research regarding e-learning [12].

Although many web-based programs have been developed in recent years for academic education in Iran (e.g. Distance Learning Network in Tehran University of Medical Sciences [DLN-TUMS] and Continuing Medical Education in Tehran University of Medical Sciences [CME-TUMS]), their quality and effectiveness have not been thoroughly and extensively evaluated [13,14]. Thus, little knowledge exists concerning web-based continuing education outcomes in Iran, and more research is clearly necessary. The aim of this study was to compare the effectiveness of web-based and face-to-face methods in continuing education about AIDS for nurses. Information about web-based education outcomes in comparison with face-to-face format can help researchers and tutors prepare and deliver future web-based or face-to-face courses more efficiently.

## Methods

### Study questions

The research questions were:

• How much did the nurses know about AIDS before and after participating in the courses?

• Is there any difference in the effectiveness of web-based instruction and face-to-face instruction in terms of the nurses' learning outcomes?

### Sample

Of 2391 nurses affiliated to the Tehran Medical University who were working in different hospitals, 140 eligible nurses were selected by the systematic random sampling method. To ensure equal numbers of patients in each group, the blocking random allocation method was used. The inclusion criteria were access to the Internet and having the necessary skills to use it, as reported by the subjects. The study was conducted in winter 2007.

### Data collection

Data were collected via two questionnaires drawn up by the researchers concerning demographic information and knowledge about AIDS. The demographic questionnaire included questions about age, gender, years of working, the current working ward, and marital status. The knowledge questionnaire consisted of 24 multiple choice questions. Correct answers scored 1 and incorrect or no-response answers scored 0. The total score of knowledge ranged from 0 to 24, which was categorized into good, moderate and poor categories on the following basis: nurses who answered fewer than 11 items correctly were classified into the "poor knowledge" class, those who answered 12 to 18 items correctly into the "moderate knowledge" class, and those who answered more than 19 items correctly into the "good knowledge" class.

The content and face validity of the knowledge questionnaire were established by a panel of experts in the field of medical-surgical nursing education. The reliability of the questionnaire was determined by the test-retest method with a 2-week interval. Twenty nurses were recruited for this purpose, who were excluded from the final analysis. Finally, a Pearson product-moment correlation coefficient of 0.90 was obtained.

### Design

A quasi-experimental method was used with a pre-test and post-test design. In both groups the key learning outcomes included the following.

On completion, the learner will be able to:

1. Describe the transmission routes of HIV infection

2. Explain the clinical manifestations of HIV infection and AIDS

3. Describe the diagnostic tests for detecting HIV infection

4. Describe the management of patients with HIV infection and AIDS

5. Describe the precautionary measures against the transmission of HIV infection

6. explain the nursing care of patients with HIV infection and AIDS

The contents of the learning materials were the same for both groups and were reviewed prior to use by a panel of medical-surgical nursing instructors who were not involved in the study. The course material was the same for both groups, and consisted of information about AIDS pathology, clinical manifestations, diagnostic tests, transmission routes, preventive measures, medical management, and nursing care. The online course consisted of a self-study text and some interactive multiple choice questions about an HIV-positive case. The participants were asked to study the online educational materials and use the interactive questions for better understanding. If they gave wrong answers to the interactive questions, a warning message led them to another one. However, if the participants selected the right answer, they received detailed information about that question. Before starting the online course, a 1-day workshop about using on-line programs and how to enter and work with the online system and chat rooms was held for the web-based group. At the beginning of the workshop, participants completed the demographic and knowledge questionnaires and each one was given a username and password. Participants were allowed to access the educational material for one week and study it at their own pace any time during day or night. In order to have interaction between the tutor and learners, and between the learners themselves, a chat room was used for synchronous and asynchronous discussion. E-mailing, telephone, and chat rooms were also used for answering questions. After one week, the passwords were expired and the online course material was not accessible anymore. Then, the knowledge questionnaire was emailed to the participants, who were asked to complete and deliver it via e-mail or in printed format. We also included these two questions in the post-test questionnaire: "how many hours did it take you to complete the online course?" and "which educational method do you prefer for future continuing education programs, a traditional face-to-face method or a web-based one?" The role of the tutor in the web-based program was managing the aforementioned workshop, preparing the course material and making it available online, receiving and answering the participants' questions via email, telephone or chat rooms, and finally sending and receiving the knowledge questionnaire.

The face-to-face group attended an interactive 3-hour lecture about AIDS given by a tutor who was also responsible for the web-based group. The participants were able to actively participate in the emerging discussions and ask their questions about the course materials and also make their class notes during the lecture. The course resources were also not available for this group after the lecture session. Then, the participants were asked to complete the post-test knowledge questionnaire. Again, we included the aforementioned question: "which educational method do you prefer for future continuing education programs a traditional face-to-face method or a web-based one?" The post-test was not open-book. Here, the tutor was responsible for preparing and presenting the course material to the participants, answering their questions, managing the lecture session, and also giving the pre- and post-tests.

### Data analysis

Data were analyzed using SPSS software version 11. Descriptive statistics involving tables of frequencies, percentages and appropriate summary statistics were used to asses the nurses' knowledge in both groups about AIDS. The independent t test was used to compare the means of knowledge in the pre- and post tests between groups. The paired t test was used to compare the means of knowledge in the pre- and post test within groups. The independent t tests and Chi-square analysis were used to compare the two groups regarding demographic characteristics. For establishing the test-retest reliability of the knowledge questionnaire, the Pearson product moment correlation test was used. The level of significance was set at p < 0.05.

### Ethics

Approval was obtained from the Ethical Review Committee of Tehran Medical Sciences University in spring 2007. Detailed information on the purpose of the study and guarantees of anonymity and confidentiality were explicitly explained to each enrollee. Written informed consent was obtained from all participants.

## Results

The average age of nurses was 33 ± 6 years in the web-base group and 33 ± 5.4 years in the face-to-face group. Most of the nurses (86.6%, n = 62, in the web-based group and 81.4%, n = 63 in the face-to-face group) were women. Most of the nurses in both groups had been working for 5–10 years and worked in internal medicine wards such as oncology or haematology. There were no statistical differences between the two groups with respect to demographic characteristics (*p *> 0.05) (Table 1).

**Table 1:** Demographic characteristics of web-based and face-to-face groups

	**Web-based group(n = 70)** **%(n)**	**Face-to-face group(n = 70)** **%(n)**	p-value*
**Age**			

20–30	32.9(23)	32.9(23)	NS
	
31–40	55.7(39)	58.6(41)	
	
41–50	8.6(6)	11.4(8)	

**Sex**			

Female	81.4(57)	86.6(62)	NS
	
Male	18.6(13)	11.4(8)	

**Years of working**			

<5	30(21)	28.6(20)	NS
	
5–10	42.8(30)	41.4(29)	
	
>10	27.14(19)	30(21)	

**Type of Ward**			

Surgical	32.9(23)	27.1(19)	NS
	
Internal	42.9(30)	40(28)	
	
Critical	24.3(17)	32.9(23)	

**Marital Status**			

Single	42.9(30)	54.3(38)	NS
	
Married	57.1(40)	45.7(32)	

* All comparisons are non-significant at p < 0.05

Half (50%, n = 35) of the web-based group and 53% (n = 37) of the face-to-face group had cared for HIV patients. In the web-based group about half (n = 35) of the nurses evaluated their computer skills as moderate, 33 as good, and 3 as poor. In this group 42 nurses connected to the internet at home, 20 nurses connected at their workplace, and eight used both.

In the web-based group, more than two thirds (70%, n = 49) of the nurses reported that they spent 5–8 hours to complete the course, compared with less than one third (30%, n = 21) who reported spending less than 5 hours. In the face-to-face group, the time spent was 4 hours. Nurses in the web-based group, on average, spent more time completing the course than those in the face-to-face group.

Forty percent of the nurses in the web-based group and 54.3% in the face-to-face group had poor knowledge about AIDS before the course, whereas most of the nurses in both groups (98.6% for the web-based and 97.1% for the face-to-face groups) had good knowledge after completing the courses.

The results show that there was no significant difference between the groups in either the pre-test (t_(138) _= -1.7, *p *= 0.096) nor the post-test (t_(138) _= -1.4, *p *= 0.163) scores in the knowledge test. However, there was a significant difference in the pre-test and post-test scores within each group (Table 2). This means that web-based and face-to-face teaching methods were equally efficient in promoting the nurses' knowledge about AIDS. The mean of knowledge gain was 9.5 in the web-base group and 9.9 in the face-to-face group. There was no significant difference between the two groups in this regard (t_(138) _= 0.706, *p *= 0.481).

**Table 2:** Pre-test and post-test mean scores of knowledge for the web-based and face-to-face groups

**Group**	no. of nurses	Pre-test Mean(SD*)	Post-test Mean(SD*)	t	*p*-value
**Web-based**	70	12.9(2.5)	22.4(1.9)	26	<0.001

**Face-to-face**	70	12.1(3.1)	22(1.6)	24.3	<0.001

*Standard Deviation

As mentioned, we included a question in the post-test questionnaire regarding the participants' preference about the teaching method to be used in future continuing education programs. An interesting finding regarding this question was that all (100%) of the nurses in the web-based and 97% in the face-to-face groups reported that they would like to take part in more continuing education using the same method.

## Discussion

The aim of this study was to compare the effectiveness of web-based and face-to-face continuing education methods in increasing nurses' knowledge about AIDS. Results suggest that these methods were equally effective: the knowledge of the nurses in both courses increased between the pre-test and the post-test, most of them scoring 'good' in the post-test. Our results are consistent with those of most other studies in finding no differences between web-based and face-to-face teaching methods regarding learning outcomes [15-19].

We also found that nurses in the web-based group, on average, spent more time completing the course than those in the face-to-face group. One possible reason for this is that the online method of instruction places more responsibility for learning on the learners[20]. Another reason may be that online learning is a new experience for many nurses in Iran. This issue could be resolved by longer training programs to give learners the opportunity to adapt to the technology[21].

Another finding was that all of the nurses in the web-based and most in the face-to-face groups reported they would like to take part in more continuing education using the same method. In the web-based group this may be related to features of web-based learning such as being self-paced and letting learners review topics when they want to[22]. But the willingness of learners in the face-to-face group to take part in continuing education using the same method may be due to the fact that they did not have much experience of web-based learning and were afraid of technology [23].

Nearly half of the nurses in each group had poor knowledge of AIDS in the pre-test, which shows that there is a need to improve the AIDS content in the nursing curriculum and to deliver continuing education about AIDS to nurses in the workplace.

There are some limitations in this study. First, although the participants in the web-based group were required to answer the post-test questions in less than 30 minutes, it was not possible to monitor the exact amount of time that they spent on completing the post-test. Moreover, as a result of technical problems we could not verify the amount of time that the nurses devoted to studying the course. We therefore had to rely on self reports of the logged-on time. Secondly, we used the same knowledge questionnaire for the pre-test and post-test, which might have caused a subject sensitization bias especially in the face-to-face group. Thirdly, as the time interval between pre-test and post-test for the face-to-face and web-based groups was not equal, bias is possible. Fourthly, the participants in the web-based group might have had access to additional educational resources about the course material. Last but not least, we also had a slow internet speed, so the nurses could not study at their desired speed, which may have meant that they would have completed the course in less time with a faster connection. A few system crashes on the first day of the course delivery were also happened, but these were quickly fixed so the nurses did not lose much time.

## Conclusion

This study found that the web-based method is as effective as the face-to-face method. This suggests that some topics in continuing education can be effectively taught via web-based courses. The web-based method is flexible, accessible (24-hour access to courses), as effective as traditional modes of teaching, convenient, self-paced and interactive. Also, developers can revise web-based course whenever the content is out-of-date, and there is no need to purchase upgrades to access web-based instruction. For these reasons, the modality will become increasingly useful to organizers of continuing education as the number of nursing staff increases. Further research should focus on the factors that contribute to optimal web-based learning. Future studies should also examine the benefits of web-based method in teaching other topics in nursing.

## Competing interests

The authors declare that they have no competing interests.

## Authors' contributions

ARK: designed the study, collected and analyzed the data and wrote the paper. NDN: was the corresponding author and the chief supervisor of the project; she co-analyzed the data and revised the drafts. FA: was a co- supervised the project and revised the drafts. HH: participated in the statistical analysis. KVJ: revised the paper, and read and commented on the drafts. All authors read and approved the final manuscript.

## Pre-publication history

The pre-publication history for this paper can be accessed here:


